# CRISPR/Cas9-Mediated Editing of *Autophagy Gene 6* in Petunia Decreases Flower Longevity, Seed Yield, and Phosphorus Remobilization by Accelerating Ethylene Production and Senescence-Related Gene Expression

**DOI:** 10.3389/fpls.2022.840218

**Published:** 2022-04-26

**Authors:** Yiyun Lin, Michelle L. Jones

**Affiliations:** Department of Horticulture and Crop Science, The Ohio State University, Wooster, OH, United States

**Keywords:** Beclin1, CRISPR, nutrient recycling, programmed cell death, petal senescence

## Abstract

Developmental petal senescence is a type of programmed cell death (PCD), during which the production of ethylene is induced, the expression of PCD-related genes is upregulated, and nutrients are recycled. Autophagy is an intracellular mechanism involved in PCD modulation and nutrient cycling. As a central component of the autophagy pathway, *Autophagy Gene 6* (*ATG6*) was previously shown as a negative regulator of petal senescence. To better understand the role of autophagy in ethylene biosynthesis and nutrient remobilization during petal senescence, we generated and characterized the knockout (KO) mutants of *PhATG6* using CRISPR/Cas9 in *Petunia* × *hybrida* ‘Mitchell Diploid.’ *PhATG6-*KO lines exhibited decreased flower longevity when compared to the flowers of the wild-type or a non-mutated regenerative line (controls), confirming the negative regulatory role of *ATG6* in petal senescence. Smaller capsules and fewer seeds per capsule were produced in the KO plants, indicating the crucial function of autophagy in seed production. Ethylene production and ethylene biosynthesis genes were upregulated earlier in the KO lines than the controls, indicating that autophagy affects flower longevity through ethylene. The transcript levels of petal PCD-related genes, including *PhATG6*, *PhATG8d*, *PhPI3K* (*Phosphatidylinositol 3-Kinase*), and a metacaspase gene *PhMC1*, were upregulated earlier in the corollas of *PhATG6-*KO lines, which supported the accelerated PCD in the KO plants. The remobilization of phosphorus was reduced in the KO lines, showing that nutrient recycling was compromised. Our study demonstrated the important role of autophagy in flower lifespan and seed production and supported the interactions between autophagy and various regulatory factors during developmental petal senescence.

## Introduction

The senescence of flower petals is a gradual process of programmed cell death (PCD) (Rogers, 2006). Based on the morphological changes, petal senescence can be categorized as wilting, withering, or abscission of turgid petals (Van Doorn, 2001). In petunia (*Petunia* × *hybrida*), the flower petals are fused to form a corolla, which wilts during senescence (Jones et al., 2009). Petal senescence can be induced by different factors, including aging, pollination, abiotic stresses, and pathogens, where different forms of PCD are involved (Van Doorn and Woltering, 2008). A sequence of events that includes nuclear fragmentation, protein degradation, nutrient mobilization, and membrane leakage accompanies petal senescence (Jones et al., 2009). Various senescence-related genes are upregulated during petal senescence, including genes involved in macromolecule degradation, nutrient remobilization, hormone biosynthesis and signaling, and cell death regulation (Broderick et al., 2014; Tsanakas et al., 2014; Wang et al., 2018; Yang et al., 2019).

Autophagy is an intracellular process that is responsible for the transportation and degradation of toxic or damaged cellular components in eukaryotic cells (Su et al., 2020). Autophagy assists in maintaining cell homeostasis and modulates developmental PCD in plants (Üstün et al., 2017). The most prominent and well-studied autophagy pathway is macroautophagy (hitherto referred to as “autophagy”), where the cellular components are enclosed in double-membrane vesicles (i.e., autophagosomes) to be transported to the vacuole for degradation (Feng et al., 2014). Autophagosomes are constructed collaboratively by proteins encoded from a variety of autophagy genes (ATGs) (Su et al., 2020). ATGs are involved in the regulation of PCD during petal senescence (Shibuya, 2012). The expression of ATGs, including *ATG1*, *ATG4*, *ATG5*, *ATG6*, *ATG7*, *ATG8a*, *b*, *d*-*f*, *ATG13*, and *Phosphatidylinositol 3-Kinase* (*PI3K*), is upregulated during petal senescence in petunia, Japanese morning glory (*Ipomoea nil*), and hibiscus (*Hibiscus rosa-sinensis*) (Shibuya et al., 2011; Broderick et al., 2014; Trivellini et al., 2016; Quijia Pillajo et al., 2018). In our previous study, silencing *ATG6* or *PI3K* in petunia results in accelerated petal senescence, as well as reduced flower number and biomass (Lin and Jones, 2021).

ATG6/VPS30/Beclin1 is a central component of the autophagy pathway and is involved in the regulation of PCD (Cao and Klionsky, 2007; Menon and Dhamija, 2018). *ATG6* is a single copy gene in Arabidopsis (*Arabidopsis thaliana*), barley (*Hordeum vulgare*), and grape (*Vitis vinifera*), as well as tobacco (*Nicotiana benthamiana*) and tomato (*Solanum lycopersicum*), which are in the same family (Solanaceae) as petunia (Tang and Bassham, 2018). Although the copy number of *ATG6* in petunia has not been confirmed, only one ATG6-like protein was found when blasting the petunia genomes with Arabidopsis *ATG6* sequence.^1^ In addition to its function in petal senescence, *ATG6* also plays an important role in the regulation of leaf developmental and stress-induced senescence. Barley leaves with silenced *ATG6* are more susceptible to darkness, oxidative stress, nutrient deficiency, and salt stress (Zeng et al., 2017). Arabidopsis, tobacco, and wheat (*Triticum aestivum*) with suppressed *ATG6* show early leaf senescence under normal conditions and enhanced pathogen-induced PCD in leaves when infected with viral, bacterial, or fungal pathogens (Liu et al., 2005; Patel and Dinesh-Kumar, 2008; Yue et al., 2015). Even though most of the studies of *ATG6* in plants focus on leaf senescence, similarities have been identified between leaf and petal senescence, including the upregulation of autophagy genes (Price et al., 2008; Wagstaff et al., 2009). Understanding the regulatory mechanisms of leaf senescence can facilitate research on petal senescence, and vice versa.

Ethylene interacts with autophagy during petal senescence (Liao and Bassham, 2020). In ethylene-treated petunia flowers, the expression of *ATG8a-d* increases, while the application of an ethylene inhibitor suppresses *ATG8a-d* expression (Shibuya et al., 2013). Treating pollinated flowers with an ethylene inhibitor delays the induced expression of *ATG8a-d* (Shibuya et al., 2013). Ethylene-induced ATG expression in flowers is thought to be regulated by ethylene-responsive transcription factors (TF). Arabidopsis ethylene-responsive TFs EIL and AP2 interact with the promoters of *ATG8a* and *ATG8h* in yeast one-hybrid assays (Wang et al., 2020). In tomato, direct binding of ethylene-responsive TF ERF5 to the promoters of *ATG8d* and *ATG18h* has been shown *in vitro* (Zhu et al., 2018). Correlations are also found between autophagy and ethylene biosynthesis. The expression of a key ethylene biosynthesis gene, 1-aminocyclopropane-1-carboxylic acid synthase (*ACC synthase*, *ACS*), decreases in the senescing petals of *ATG6*- or *PI3K*-silenced petunias (Lin and Jones, 2021). In *PI3K*-overexpressing tobacco, the production of ethylene is enhanced as a result of increased expression of another ethylene biosynthesis gene *ACC oxidase 1* (*ACO1*) despite decreases in the expression of *ACO2* (Dek et al., 2017).

The remobilization of mineral nutrients is a critical process during petal senescence (Jones, 2013). Evolutionarily, the purpose of flower petals is to attract pollinators for sexual reproduction. Because it is energetically costly to maintain these elaborate structures, the flower petals senesce once the flower is pollinated, or when the stigma is no longer receptive to pollination (Jones et al., 2009). To preserve resources, nutrients are remobilized from the senescing petals to other tissues or organs for recycling (Borghi and Fernie, 2020). Nutrient remobilization during petal senescence is regulated by autophagy (Avila-Ospina et al., 2014). Nuclear fragmentation and DNA mass reduction are delayed in pollinated flowers treated with 3-methyladenine (3-MA), an inhibitor of autophagy protein PI3K (Yamada et al., 2009). The application of the autophagy inhibitor concanamycin A limits the growth of the ovary in pollinated flowers, suggesting that autophagy is a key regulator in pollination-mediated nutrient recycling (Shibuya et al., 2013). Phosphorus (P) and nitrogen (N) are two of the major nutrient elements remobilized during petal senescence (Jones, 2013), and an increase of N and P is found in the ovary as a sink organ after pollination-induced senescence (Hew et al., 1989). Ethylene plays an important role in autophagy-mediated nutrient remobilization during petal senescence (Jones, 2013). In both pollinated and unpollinated senescing flowers, the remobilization of N and P is reduced in ethylene-insensitive transgenic petunia compared to the ethylene-sensitive wild-type control (Jones, 2008).

The clustered regularly interspaced short palindromic repeats (CRISPR) and associated endonuclease 9 (Cas9) system is a powerful tool for reverse genetic analysis and horticulture crop improvement (Karkute et al., 2017). Mutagenesis of the *ACO* genes in petunia or a NAC transcription factor in Japanese morning glory using CRISPR/Cas9 successfully delays petal senescence in these plants (Shibuya et al., 2018; Xu et al., 2020, 2021). To better understand the function of *PhATG6* in the regulation of corolla senescence and nutrient recycling in petunia and to characterize ethylene’s role in autophagy-mediated corolla senescence, we analyzed stable *PhATG6*-knockout (KO) mutants generated with CRISPR/Cas9-mediated gene editing.

## Materials and Methods

### Plant Materials

Seeds of petunia (*Petunia* × *hybrida*) inbred line ‘Mitchell Diploid’ (MD) were surface-sterilized with 95% ethanol for 1 min and 10% bleach for 10 min before rinsing with sterile water three times. The sterilized seeds were sown on ½ strength MS (Murashige and Skoog) media to generate sterile seedlings. Seedlings were grown under a 16-h light/8-h dark cycle at 25°C for 4–7 weeks until the leaves were ready to be used for transformation.

### Plasmid Construction

**Figure 1:**
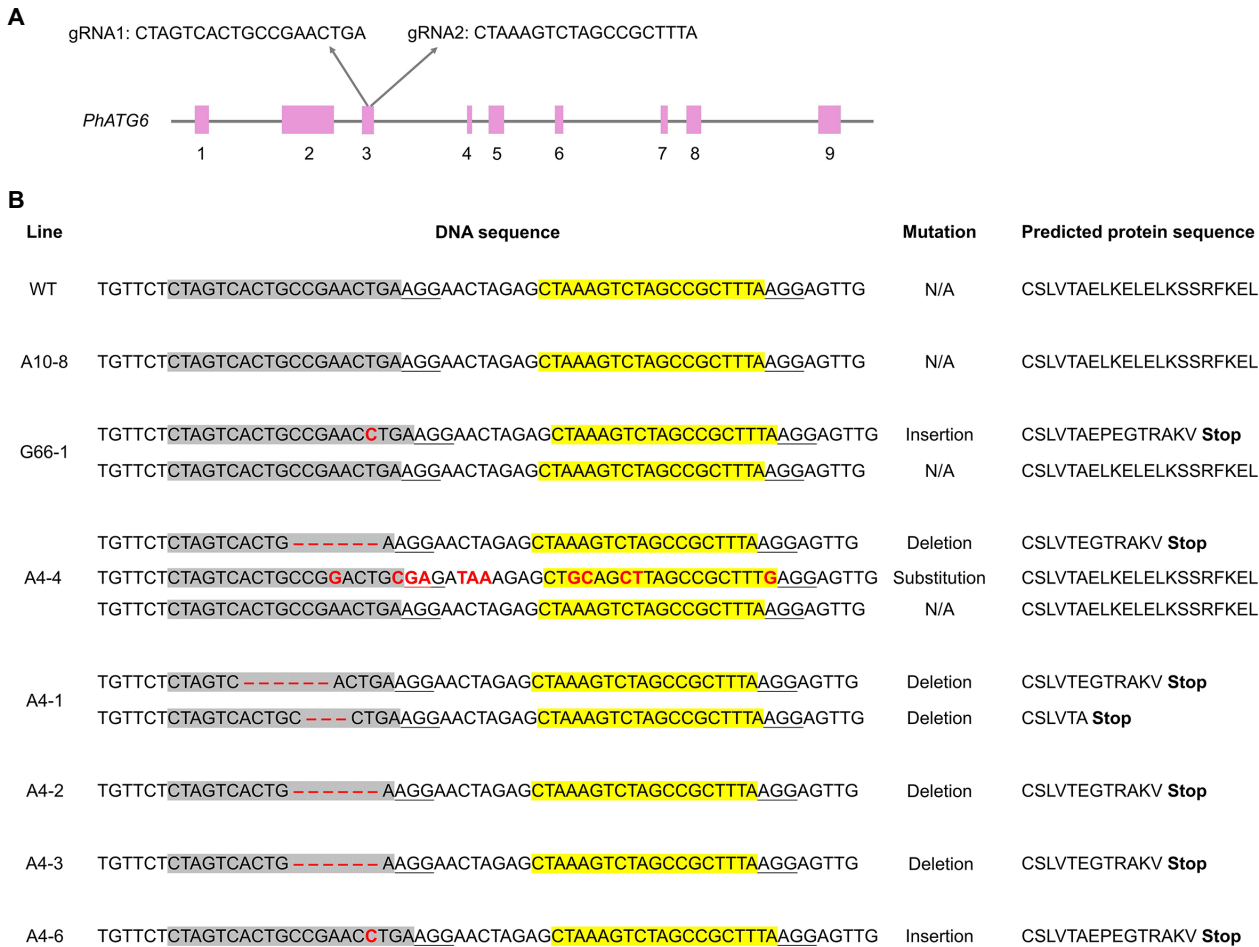
CRISPR/Cas9-edited sequences of *Autophagy Gene 6* (*PhATG6*) in T_0_ *Petunia* × *hybrida* ‘Mitchell Diploid’ plants with mutated *PhATG6* and wild-type control (WT). **(A)** Two guide RNAs (gRNAs) were used to target *PhATG6* exon 3. **(B)** DNA sequences and predicted protein sequences of *PhATG6* exon 3 in wild-type plant, non-mutated regenerated line A10-8, and different *PhATG6*-KO lines. Pink boxes represent exons 1–9 of *PhATG6*, grey sequences represent the targeted site of gRNA1, yellow sequences represent the targeted site of gRNA2, and red texts represent mutation sites. The protospacer adjacent motif (PAM) for Cas9 recognition (NGG) was underlined. N/A represents no mutation detected.

The CRISPR plasmid for generating the *PhATG6* knockout (KO) was constructed following a published protocol (Xing et al., 2014). The vector pKSE401 was from Qi-Jun Chen laboratory (plasmid #62202; Addgene, Watertown, MA, United States). This binary vector contains the sequence of *Cas9* and an insert site for gRNA sequences, along with a kanamycin resistance selective marker. The genomic DNA sequence of *PhATG6* (Subject ID: Peaxi162Scf00403) was obtained from the petunia genome sequence in the Sol Genome Network^2^ for gRNA design. Specifically, 23-bp target sites (including a 20-bp gRNA sequence followed by a 3-bp sequence NGG, a PAM for Cas9) were identified manually within the exons of the *PhATG6* DNA sequence as potential gRNAs. These potential gRNA sequences were evaluated for the potential knockout accuracy^3^, and two gRNAs were selected from exon 3 of *PhATG6* DNA (Figure 1). The gRNAs were inserted into the pKSE401 vector under an AtU6-26 promoter using a Golden Gate Assembly method (Xing et al., 2014).

### Generation of *PhATG6*-KO Lines

The constructed plasmids carrying both *Cas9* and *PhATG6* gRNA sequences were introduced into *Agrobacterium tumefaciens* GV3101 *via* electroporation for *Agrobacterium*-mediated plant transformation. The transformation was carried out using an established protocol modified from Jorgensen et al., 1996. Briefly, *Agrobacterium* was cultured on an LB plate with selective antibiotics kanamycin (50 mg L^−1^), gentamicin (25 mg L^−1^), and rifampicin (10 mg L^−1^). Single colonies were selected to culture in 6-ml liquid LB media with the same antibiotics overnight at 28°C. The overnight culture was used to inoculate 50 ml selective LB media at a 1:10 ratio and cultured at 28°C until OD_600_ = 0.8–1.0. The culture was diluted to OD_600_ = 0.15 with co-culture MS media including 6-benzylaminopurine (BAP, 2 mg L^−1^), indole-3-butyric acid (IBA, 5 μg L^−1^), and acetosyringone (0.2 mM). Young, fully expanded leaves were excised from petunia plants and cut into 5 × 5 mm^2^ pieces. Leaf explants were immersed in diluted *Agrobacterium* culture for 15 min, blotted dry using sterile filter paper, and placed on co-culture medium plates with the adaxial side facing down. The medium plates were sealed and incubated in the dark at room temperature for 3 days. After that, the explants were transferred and cultured on regeneration media (MS with 2 mg L^−1^ BAP, 5 μg L^−1^ IBA, 150 mg L^−1^ kanamycin, and 500 mg L^−1^ carbenicillin) to induce callus and shoots. The regenerated shoots were excised from the base without any callus and transferred to rooting media (MS with 150 mg L^−1^ kanamycin and 500 mg L^−1^ carbenicillin) for root induction.

### Validation of *PhATG6*-KO Lines

Once the transformed plants were regenerated through tissue culture, DNA sequencing was used to verify whether the *PhATG6* gene was mutated. Total DNA from each event was extracted from leaf tissue using a DNeasy kit (Qiagen, Hilden, Germany). Fragments of the *PhATG6* sequences were amplified *via* PCR, and the PCR products were cloned into a sequencing vector pJET1.2 using CloneJET PCR Cloning Kit (Thermo Fisher Scientific, Waltham, MA, United States). The cloned products were transformed into chemically competent *Escherichia coli* using the Mix & Go *E. coli* Transformation Kit (Zymo Research, Irvine, CA, United States), and the transformation was verified with colony PCR. At least five colonies from each event were sent to Molecular Cloning Laboratories (MCLAB, South San Francisco, CA, United States) for sequencing to identify the specific *PhATG6* gene edits. The DNA sequences of the mutated genes were used to determine the predicted protein sequences using Expasy Translate.^4^ Plants with mutated *PhATG6* are named *PhATG6*-KO lines hereinafter, and a line identified without any mutation in *PhATG6* is referred to as the non-mutated line.

### Plant Phenotypic Evaluation

The regenerated transgenic plantlets were moved out of *in vitro* culture and transplanted into 6.4-cm pots with a peat-based potting mix (Promix BX; Premier Tech Horticulture, Quebec, Canada). Plants were placed in a clear bin with a lid that was opened over time to slowly acclimate the plants. The acclimated plants were moved into a greenhouse at The Ohio State University CFAES Wooster Campus (Wooster, OH, United States). Wild-type MD petunia seeds were sown in a peat-based germination mix (Promix PGX; Premier Tech Horticulture). Four-week-old seedlings were transplanted to 6.4-cm pots with a peat-based media (Promix BX; Premier Tech Horticulture) and moved into the greenhouse with the regenerated plants. One cutting was made from each of the mutants and the wild-type plant, and the new plants produced from the cuttings were transplanted into 3.8-L pots with the same peat-based media (Promix BX; Premier Tech Horticulture) and grown under 23°C day/18°C night temperature with 14-h light/10-h dark cycle. Plants were fertigated with 75 mg L^−1^ N from 15N-2.2P-12.5K-2.9Ca-1.2Mg water-soluble fertilizer (Jack’s Professional LX, JR Peters, Allentown, PA, United States).

The phenotypic evaluation of all plants included measurements of flower longevity, flower size, capsule weight, capsule maturation time, number of seeds per capsule, and 100-seed weight. To measure the longevity of flowers in planta, the anthers were removed from the flowers 1 day before opening (deanthered), and the dates of corolla opening and senescence were recorded. Flower longevity (*n* = 10) was calculated as the number of days from corolla opening to when it senesced (wilted but not dry). Flower size (*n* = 4) was determined by measuring the diameters of flowers using a ruler on the day of opening. To collect capsules, the anthers were removed from the flowers 1 day before opening, the flowers were pollinated with pollen from the same plant on the day of opening, and capsules were collected when they were mature (the capsules were brown and dried). The average number of days from flower pollination to capsule collection was recorded as capsule maturation time. Five-to-eight mature capsules were collected from each plant, placed in a 1.5-ml centrifuge tube, and dried in a chamber with desiccant for 1 month. The seeds in each capsule were isolated and counted. The weight of the dry capsules and 100-seed weight was measured using a balance (Adventurer Pro AV313; Ohaus, Pine Brook, NJ, United States).

### Evans Blue Staining

The corolla limbs of the flowers were stained with Evans Blue (MP Biomedicals, Solon, OH, United States), a dye used to stain dead cells. The deanthered flowers were collected daily from the day before flower opening (day −1) to the day after the last day of senescence. Individual petal limbs were excised and submerged in Evans Blue solution (0.1% *w*/*v*) for 20 min, rinsed with distilled water three times, and blotted dry with filter paper. Photographs were taken immediately after the staining process.

### Ethylene Production Measurements

The production of ethylene was measured using a Varian CP-3800 gas chromatograph (GC) equipped with a flame ionization detector (FID) and a HayeSep R packed column (Agilent Technologies, Santa Clara, CA, United States) following a previously published method (Broderick et al., 2014). The deanthered flowers were collected on day 0 and day 3 after flower opening, and daily starting on day 6 until the last day of senescence. The whole corollas were isolated and individually incubated in sealed 22-ml glass vials for 2 h. From each vial, a 1-ml gas sample was withdrawn and injected into the GC to determine ethylene concentration (nl). Ethylene production was calculated by dividing the ethylene concentration by the fresh weight of the corolla (g) and the incubation time (h). Four-to-six corollas were analyzed for each line at each time point.

### Gene Expression Analysis

The expression of senescence-related genes in corollas was measured following a previously reported method (Lin and Jones, 2021). Briefly, deanthered flowers were collected daily from flower opening (day 0) through senescence from different plants, and the corollas were immediately frozen using liquid nitrogen and stored at −80°C. Total RNA was extracted from three finely ground corollas using TRIzol Reagent (Invitrogen, Carlsbad, CA, United States) and treated with RQ1 RNase-free DNase (Promega, Madison, WI, United States). A spectrophotometer (NanoDrop ND-1000; Thermo Fisher Scientific) was used to determine the concentration of RNA. Gene expression was measured *via* RT-qPCR (reverse transcription-quantitative PCR) using cDNA synthesized from 1 μg RNA with iScript Reverse Transcription Supermix (Bio-Rad, Hercules, CA, United States). The qPCR was conducted using SsoAdvanced Universal SYBR Green Supermix (Bio-Rad) in a C1000 Touch™ Thermo Cycler (Bio-Rad). The procedure for qPCR was 30 s at 95°C for denaturation, 40 cycles of 10 s denaturation at 95°C and 30 s annealing and extension at 50°C, followed by melt curve analysis. The relative expression was calculated using CFX Manager™ software (Bio-Rad). Both *PhActin* (Chapin and Jones, 2009) and *PhSAND* (Mallona et al., 2010) were used as reference genes. Primers used for qPCR are listed in Supplementary Table S1. Two biological replicates and two technical replicates were included for each gene expression analysis.

### Flower Tissue Nutrient Analysis

Flower corollas were collected on the day of flower opening (non-senescing) and the last day of flower senescence (senescing). The samples were dried in a forced air-drying oven set at 60°C, ground into a powder that could pass a 2-mm sieve, and sent to the Service Testing and Research (STAR) Lab (The Ohio State University, Wooster, OH, United States) for nutrient analysis. Total nitrogen (N) was analyzed using a Vario Max combustion analyzer (Elementar Americas, Ronkonkoma, NY, United States) following the Dumas combustion method (Sweeney, 1989). Plant tissue was digested using an automated microwave digestion system (Discover SP-D, CEM, Matthews, NC, United States). The concentrations of other mineral nutrients including phosphorus (P) were determined using an inductively coupled plasma spectrometer (Agilent 5110 ICP-OES, Agilent Technologies, Santa Clara, CA, United States; Isaac and Johnson, 1985). The remobilized nutrients were determined by subtracting the nutrient concentrations that remained in the senescing corollas from the concentrations in the non-senescing corollas. Three replicates were included for each line at each time point.

### Statistical Analysis

All statistical analyses were conducted following the model y = μ + Line + Replicate + e in R 3.3.1 (R Core Team, 2013). All the *PhATG6*-KO lines and the non-mutated regenerated line A10-8 were compared to the wild-type control using ANOVA-protected two-sided Dunnett’s test (multcomp package).

## Results

### *PhATG6* Was Knocked Out Using CRISPR/Cas9

To understand the function of *PhATG6*, CRISPR/Cas9 was used to induce *PhATG6* editing in petunia. Based on the DNA sequences of exon 3 of the *PhATG6* gene in the wild-type and the regenerated plants, we identified 13 *PhATG6*-KO lines with mutated *PhATG6* out of 23 regenerated plants, resulting in a mutation frequency of 57% (Table 1). Seven biallelic mutants, where both alleles of *PhATG6* were mutated, and two monoallelic mutants, where only one allele of *PhATG6* was mutated, were regenerated (Table 1). In addition, we also generated four chimeric lines (Table 1). Different mutations were detected in the KO lines, including deletions, insertions, and base substitutions in *PhATG6* exon 3 (Figure 1). Based on the nucleotide sequences, the predicted amino acid sequences were determined. Lines with truncated protein sequences were considered to be loss-of-function *PhATG6* mutants (Figure 1). Further characterizations were conducted using the *PhATG6*-KO lines including four biallelic mutants (A4-1, A4-2, A4-3, A4-6) with loss-of-function *PhATG6*, 1 monoallelic mutant (G66-1), and 1 chimeric mutant (A4-4). A non-mutated regenerated line (A10-8) and a wild-type plant (WT) were used as controls (Figure 1).

**Table 1:** Validation of *PhATG6* editing.

	Total number
Number of explants	200
Number of lines regenerated	23
Number of lines with mutated *PhATG6*	13
Mutation frequency	57%
Number of biallelic mutants	7
Number of monoallelic mutants	2
Number of chimeric lines	4

### *PhATG6*-KO Lines Showed Decreased Flower Longevity

**Figure 2:**
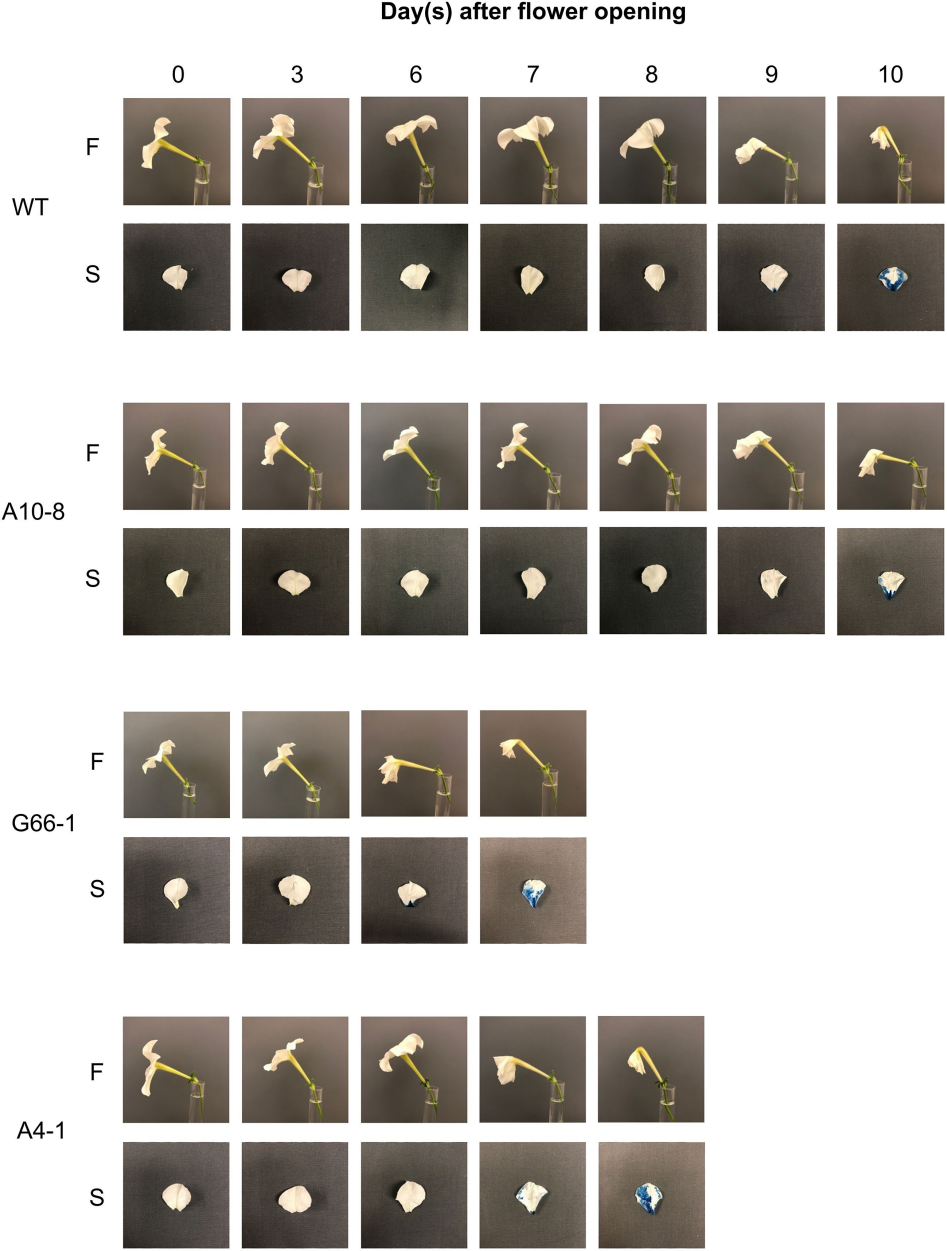
Corolla senescence in *Petunia* × *hybrida* ‘Mitchell Diploid’ wild-type plant (WT), non-mutated regenerated line A10-8, and *PhATG6*-KO lines (G66-1 and A4-1). Pictures of representative flowers (F) and Evans Blue-stained petal limbs (S) were taken from the day of flower opening (day 0) to the day of flower senescence. Additional timepoints and lines can be seen in Supplementary Figure S1.

**Figure 3:**
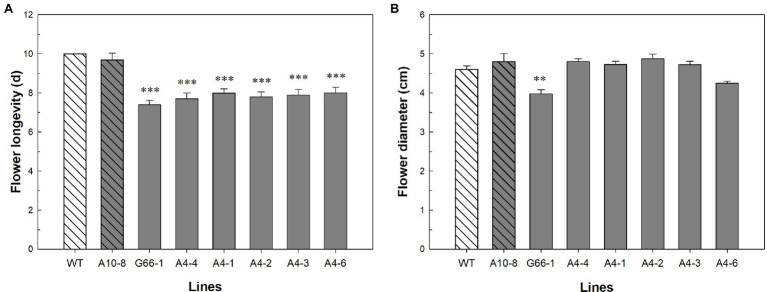
Flower phenotypic evaluation of *Petunia* × *hybrida* ‘Mitchell Diploid’ wild-type plant (WT), non-mutated regenerated line A10-8, and *PhATG6*-KO lines. **(A)** Flower longevity (*n* = 10). **(B)** Flower diameter (*n* = 4). Bars represent the means of the measurements ± standard error, and bars with stars indicate a significant difference compared to the WT: “***”*p* ≤ 0.001, “**”*p* ≤ 0.01.

Flowers of all six *PhATG6*-KO lines had reduced flower longevity (i.e., accelerated corolla senescence) when compared to the controls (Figures 2, 3A; Supplementary Figure S1). Flowers of the control plants lasted an average of 10 days, while flowers of the KO lines lasted an average of 7–8 days (Figure 3A). Photographs of the corolla senescence process showed that the senescence of petunia corollas began with the collapsing of the neck connecting the limb and the tube and then proceeded to the wilting of the limb (Figure 2; Supplementary Figure S1). Evans blue staining of the flower petal limbs also showed that cell death (loss of membrane integrity) started in the neck and continued to move up to the rest of the limb (Figure 2; Supplementary Figure S1). The corolla senescence pattern was similar in flowers of the *PhATG6*-KO lines compared to the controls (Figure 2; Supplementary Figure S1). KO line G66-1 had smaller flowers than the controls (Figure 3B). Flowers produced by line G66-1 were 17% smaller than the flowers of line A10-8 and 13% smaller than wild-type flowers (Figure 3B).

### Seed Number Decreased While Individual Seed Weight Increased in *PhATG6*-KO Lines

**Figure 4:**
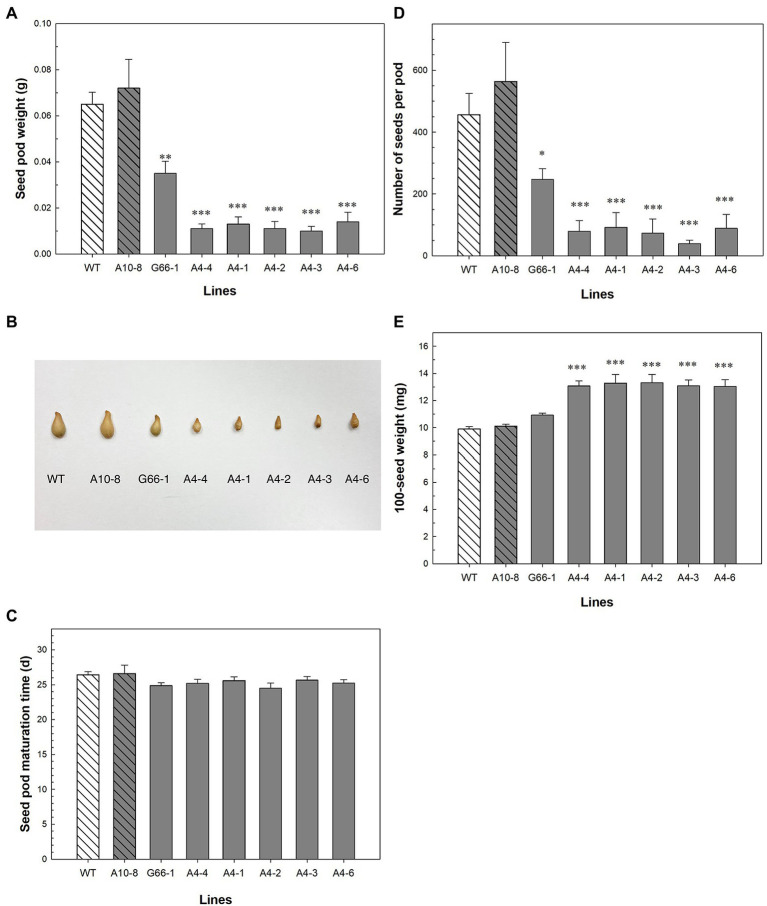
Seed phenotypic evaluation of *Petunia* × *hybrida* ‘Mitchell Diploid’ wild-type plant (WT), non-mutated regenerated line A10-8, and *PhATG6*-KO lines. **(A)** Capsule weight (*n* = 3). **(B)** Picture of representative capsules. **(C)** Capsule maturation time (*n* = 8). **(D)** Number of seeds per capsule (*n* = 3). **(E)** 100-seed weight (*n* = 4). Bars represent the means of the measurements ± standard error, and bars with stars indicate a significant difference compared to the WT: “***”*p* ≤ 0.001, “**”*p* ≤ 0.01, “*”*p* ≤ 0.05.

The production of seeds was impacted in the *PhATG6*-KO lines. The weight of seed capsules (fruits) was 46–86% lower in the KO lines compared to the controls (Figure 4A). The capsules from the KO lines were visually smaller than the control capsules (Figure 4B). There was no difference in capsule maturation time (Figure 4C). The numbers of seeds were decreased by 46–93% in the KO lines compared to the controls (Figure 4D). In contrast, the 100-seed weight increased 23–26% in five of the *PhATG6*-KO lines compared to the controls, while line G66-1 did not show any statistical difference (Figure 4E). Compared to the other five *PhATG6*-KO lines, G66-1 had a smaller difference in capsule weight, seed number per capsule, and 100-seed weight compared to the controls (Figures 4A,D,E).

### Ethylene Production Was Induced Earlier in *PhATG6*-KO Flowers

**Figure 5:**
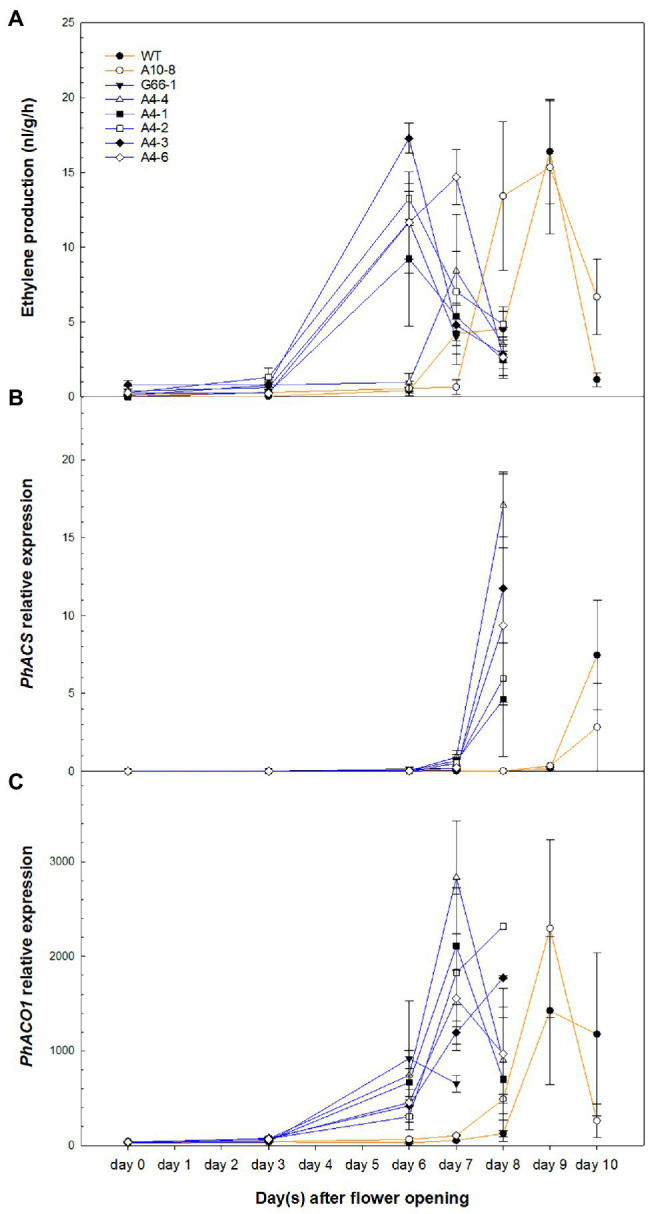
Ethylene production and ethylene biosynthesis gene expression in the corollas of *Petunia* × *hybrida* ‘Mitchell Diploid’ wild-type plant (WT), non-mutated regenerated line A10-8, and *PhATG6*-KO lines from flower opening to senescence. **(A)** Ethylene production (*n* = 6). **(B)** Relative expression of *1-Aminocyclopropane 1-Carboxylate Synthase* (*PhACS*; *n* = 2). **(C)** Relative expression of *1-Aminocyclopropane 1-Carboxylate Oxidase 1* (*PhACO1*; *n* = 2). Orange lines represent the results of controls (WT and line A10-8), and blue lines represent the results of the *PhATG6*-KO lines. The relative expression was calculated based on the expression of reference genes *PhActin* and *PhSAND*. Data represent the means of the measurements ± standard error.

To determine whether ethylene is involved in the *PhATG6-*KO-accelerated corolla senescence, the production of ethylene, as well as the transcript levels of ethylene biosynthesis genes *PhACS* and *PhACO1*, was measured overtime after flower opening. The levels of ethylene production from the corollas increased over time and reached a peak 1–2 days before the last day of senescence in the controls and the KO lines (Figure 5A). Ethylene production was induced earlier in the *PhATG6*-KO corollas concomitant with their earlier senescence (Figure 5A). The amount of ethylene produced at the peaks varied among the KO lines, but was not different than that of the controls (Figure 5A). Two ethylene biosynthesis genes in petunia, *PhACS* and *PhACO1*, were similarly upregulated earlier in the KO corollas than the control corollas, corresponding with the earlier ethylene production (Figures 5B,C). The expression of *PhACS* in flower corollas was upregulated on day 8 in the KO lines and on day 10 in the controls (Figure 5B). Variation in *PhACS* transcript levels was found across the *PhATG6*-KO lines when they reached the maximum expression (Figure 5B). The expression of *PhACO1* in corollas increased over time, reaching the maximum expression on day 7 or 8 in the KO lines, and on day 10 in the controls (Figure 5C). The maximum expression levels of *PhACO1* were also variable among the *PhATG6*-KO lines (Figure 5C).

### PCD-Related Gene Expression Was Upregulated Earlier in *PhATG6*-KO Flowers

**Figure 6:**
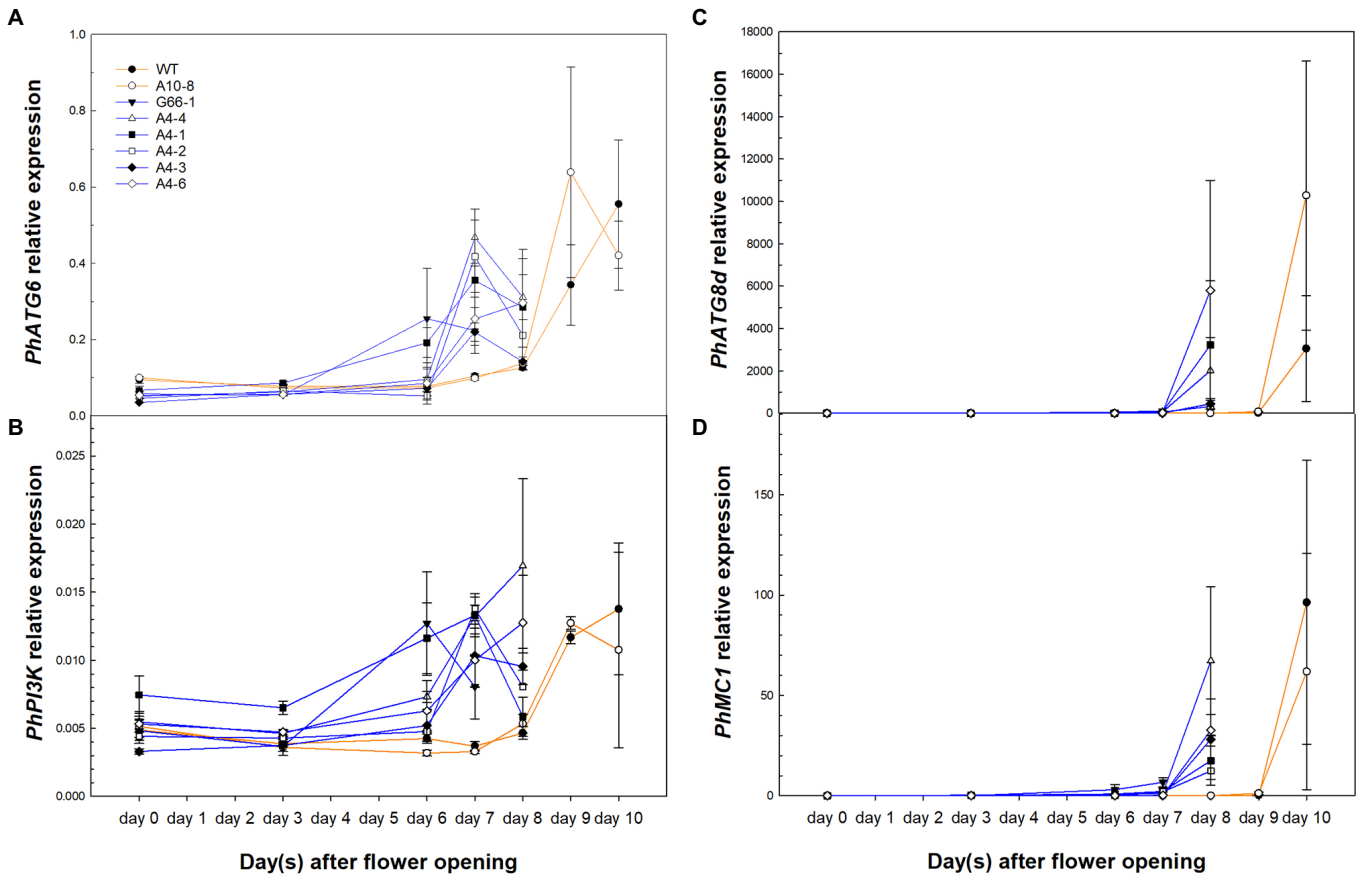
Relative expression of PCD-related genes *Autophagy Gene 6* (*PhATG6*, **A**), *Phosphoinositide 3-Kinase* (*PhPI3K*, **B**), *Autophagy Gene 8d* (*PhATG8d*, **C**), and *Metacaspase 1* (*PhMC1*, **D**) in the corollas of *Petunia* × *hybrida* ‘Mitchell Diploid’ wild-type plant (WT), non-mutated regenerated line A10-8, and *PhATG6*-KO lines from flower opening to senescence (*n* = 2). Orange lines represent the results of controls (WT and line A10-8), and blue lines represent the results of the *PhATG6*-KO lines. The relative expression was calculated based on the expression of reference genes *PhActin* and *PhSAND*. Data represent the means of the measurements ± standard error.

To determine whether knocking out *PhATG6* could affect the function of PCD-related genes in corolla senescence, the relative expression of *PhATG6*, *PhPI3K*, *PhATG8d*, and a metacaspase gene *Metacaspase 1* (*PhMC1*) was measured and found to be upregulated earlier in the KO corollas than the controls, corresponding to the time of corolla senescence (Figure 6). *PhATG6* transcript levels were upregulated on the last 2 days of the corolla senescence process in the controls and all the KO lines, with variations in the expression found across the different KO lines (Figure 6A). The relative expression of *PhPI3K* was also enhanced on the last 2 days of corolla senescence in every plant, and the transcript levels were variable among the different *PhATG6*-KO lines (Figure 6B). *PhATG8d* was upregulated on the last day of the senescence process in the corollas of the controls (day 10), and the *PhATG6*-KO plants (day 7 or 8), and the relative expression of *PhATG8d* varied among the KO lines (Figure 6C). Similarly, *PhMC1* expression was upregulated on the last day of corolla senescence, and it was induced earlier in the *PhATG6*-KO flowers (day 7 or 8) than the controls (day 10) (Figure 6D). Variations in the transcript levels of *PhMC1* were observed among the *PhATG6*-KO lines (Figure 6D).

### Nutrient Remobilization Was Impaired in *PhATG6*-KO Flowers

**Figure 7:**
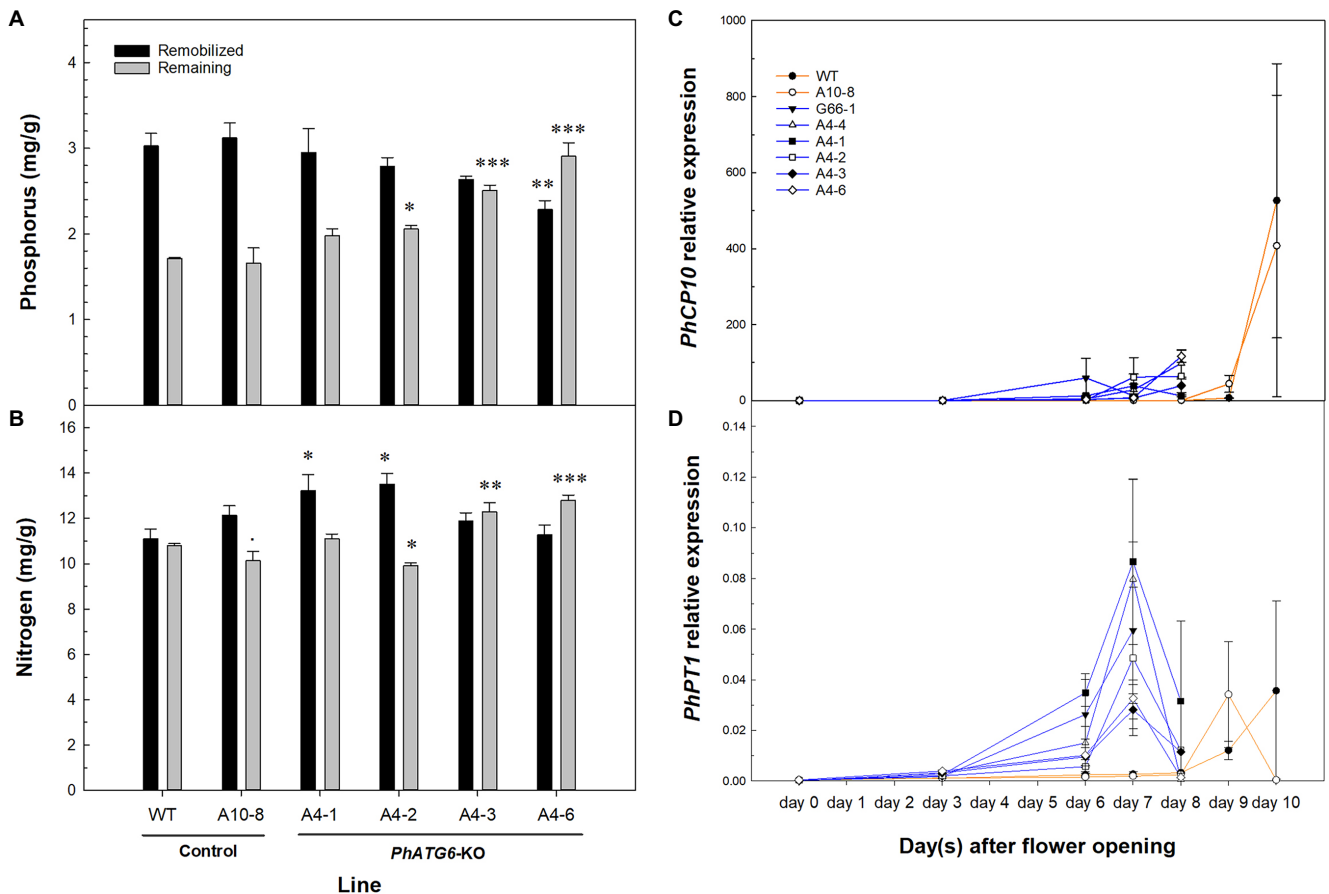
Nutrient mobilization from corollas of the *Petunia* × *hybrida* ‘Mitchell Diploid’ wild-type plant (WT), non-mutated regenerated line A10-8, and *PhATG6*-KO lines. Concentration of phosphorus (P) **(A)** and nitrogen (N) **(B)** that was remobilized (black) and remaining (grey) in the senescing corollas (*n* = 3). Relative expression of senescence-associated *Cysteine Protease 10* (*PhCP10*) **(C)** and *Phosphate Transporter 1* (*PhPT1*) **(D)** from corolla opening to senescence (*n* = 2). The remaining P and N is the concentration in senescing corollas. Remobilized N and P were calculated by subtracting the concentration of individual nutrients in the senescing corollas from the concentration in the non-senescing corollas. Orange lines represent the results of controls (WT and line A10-8), and blue lines represent the results of the *PhATG6*-KO lines. The relative expression was calculated based on the expression of reference genes *PhActin* and *PhSAND*. Data represent the means of the measurements ± standard error. Bars represent the means of the measurements ± standard error, and bars with stars indicate a significant difference compared to the WT: “***”*p* ≤ 0.001, “**”*p* ≤ 0.01, “*”*p* ≤ 0.05, “.”*p* ≤ 0.1.

To characterize the effects of *PhATG6* KO on nutrient remobilization during petal senescence, we measured the concentrations of different macro- and micro-nutrients in the corollas of KO lines and the controls. By comparing the total nutrient content of non-senescing and fully senescent corollas, the difference represents the nutrients that were remobilized from the corolla during the senescence process. The mobilization of phosphorus (P) was reduced in the *PhATG6*-KO lines compared to the controls (Figure 7A). From corolla opening to senescence, 64 and 65% of P was remobilized from the wild-type plant and line A10-8, respectively, while only 60, 58, 51, and 44% of P was remobilized from the KO lines A4-1, A4-2, A4-3, and A4-6 (Figure 7A). We did not observe reduced remobilization of nitrogen (N) or other macro-nutrients (potassium, magnesium, calcium, and sulfur), nor any micro-nutrient (aluminum, boron, copper, iron, manganese, molybdenum, and zinc) evaluated during corolla senescence in the *PhATG6*-KO lines compared to the controls (Figure 7B, data not shown). A senescence-associated cysteine protease, *PhCP10*, thought to be involved in protein degradation, was upregulated at the final stage of corolla senescence, earlier in the *PhATG6*-KO flowers than in the flowers of the controls (Figure 7C). Variations were found in the *PhCP10* transcript levels among the *PhATG6*-KO lines; however, our results showed a trend of reduced *PhCP10* expression in the KO flowers compared to the controls, even though the maximum expression levels were not statistically different (Figure 7C). The relative expression of a senescence-associated phosphate transporter *PhPT1* was also upregulated on the last 2 days of corolla senescence (Figure 7D). Variations in *PhPT1* expression were shown among the *PhATG6*-KO lines (Figure 7D).

## Discussion

Knocking out *PhATG6* in petunia accelerated corolla senescence by 2–3 days (Figure 3A), indicating that autophagy is involved in delaying corolla senescence. This result supports our previous finding, where silencing *PhATG6* using Virus-induced Gene Silencing (VIGS) results in decreased flower longevity by 3–4 days in petunia (Lin and Jones, 2021). Even though knocking out *PhATG6* with CRISPR did not seem to reduce flower longevity as much as VIGS, this could be due to the difference in the plant growing environment and petunia cultivars. The *PhATG6*-KO lines (‘Mitchell Diploid’) were grown under greenhouse conditions, while the *PhATG6*-silenced petunias (‘Picobella Blue’) were grown in growth chambers. The proliferation of virus may also play a role by placing more stress on the VIGS plants, leading to a more severe reduction of flower longevity. In addition to *ATG6*, the genetic modifications of another autophagy gene, *PI3K*, also affect the longevity of flowers (Dek et al., 2017; Lin and Jones, 2021).

The morphological cell death processes in flower corollas vary in different species. In Japanese morning glory, corolla PCD starts from the edge of the petal limbs and gradually moves toward the base of the petals (Shibuya et al., 2014). Our results showed that petunia corolla PCD began in the corolla neck connecting the limb and tube and proceeded to the rest of the corolla, with the majority of petal cells maintaining intact plasma membranes until the last 1–2 days of the senescence process (as shown by Evans Blue staining; Figure 2). Similarly, in Alstroemeria (*Alstroemeria peruviensis*), the PCD in petals does not occur until the last stages of flower senescence, as demonstrated by the electrolyte leakage, DNA laddering, and protease activity in petals (Wagstaff et al., 2003). Cut daylily (*Hemerocallis* sp.) flowers also do not show reduced membrane stability until visible senescence symptoms appear (Chakrabarty et al., 2009). Our results provide further evidence that the loss of membrane integrity does not occur gradually but at the late stage of petal senescence.

*PhATG6* is functionally involved in fruit (capsule) and seed production in petunia (Figure 4). Autophagy is the major regulator of nutrient recycling and therefore indirectly affects the nutrient supplies for seeds (Ren et al., 2014). In rice (*Oryza sativa*), the overexpression of *ATG8a* or *ATG8b* increases grain number and yield per plant, which are reduced in plants with RNAi-suppressed *ATG8b* compared to the wild-type plants (Yu et al., 2019; Fan et al., 2020). Rice *atg7* mutants and *ATG8b*-RNAi plants also show reduced grain quality, demonstrated by the chalky appearance and the starch and protein composition of the grains (Sera et al., 2019; Fan et al., 2020). Arabidopsis mutants of *ATG5* or *ATG7* produce fewer seeds per silique, fewer siliques per plant, and lower seed yield than the wild-type plants (Guiboileau et al., 2012; Barros et al., 2017; Lornac et al., 2020). Arabidopsis plants with heterozygous *PI3K* (+/−) gene produce fewer numbers of seeds per silique (Lee et al., 2008). The number of siliques per plant is decreased in Arabidopsis heterozygous *atg6* (+/−) mutants (Fujiki et al., 2007; Harrison-Lowe and Olsen, 2008). Similarly, we observed smaller capsules and fewer seeds per capsule in the *PhATG6*-KO petunias compared to the controls (Figures 4A,B,D). The average weight of individual seeds was higher in most of the *PhATG6*-KO lines than the control plants (Figure 4E), consistent with what is observed in Arabidopsis *atg7* mutants (Barros et al., 2017). Although we did not find a difference in capsule maturation time between the *PhATG6*-KO plants and the controls (Figure 4C), a study in pepper (*Capsicum annuum*) shows an increase in autophagy gene expression, autophagy protein abundance, and autophagosome-like vesicles in mature fruit, suggesting that autophagy is involved in fruit ripening (López-Vidal et al., 2020).

Ethylene is involved in the regulation of accelerated corolla senescence in the *PhATG6*-KO lines. As a regulator for senescence in plants, ethylene plays a key role in autophagy-mediated petal senescence (Shibuya et al., 2013). The early elevation of ethylene levels was likely the reason for early corolla senescence in the *PhATG6*-KO lines. Our results suggested that autophagy could affect the timing of ethylene biosynthesis. However, in tobacco plants with overexpressed *PI3K*, even though the detached flower senescence is accelerated, and the ethylene production is higher in the entire detached flowers, no difference in the timing of ethylene production is observed (Dek et al., 2017). The difference in ethylene production timing may indicate different regulatory mechanisms of corolla senescence. The expression of ethylene biosynthesis genes *PhACS* and *PhACO1* was also induced earlier in the *PhATG6*-KO corollas compared to the controls, but later than the elevated ethylene levels (Figures 5B,C). As shown in carnation flowers, ethylene treatment can induce the expression of ethylene biosynthesis genes (Jones, 2003). Therefore, the increase in ethylene biosynthesis gene expression in petunia corollas was likely induced by the earlier produced ethylene. Multiple *ACS* genes with different roles have been found in Arabidopsis, tobacco, carnation, orchid (*Phalaenopsis* sp.), tomato, and potato (*Solanum tuberosum*) (Yip et al., 1992; Destéfano-Beltrán et al., 1995; Liang et al., 1995; Bui and O’Neill, 1998; Jones and Woodson, 1999; Ge et al., 2000), while little is known about the function of additional *ACS* genes in petunia. It is possible that the ethylene produced earlier in the corollas of *PhATG6*-KO flowers was synthesized by another *ACS* member in petunia. Another possibility is that the ethylene could be synthesized using ACC translocated from other parts of the flowers, or the ethylene itself could be translocated, as shown in carnation and orchid (Reid et al., 1984; Woltering, 1990; O’Neill et al., 1993). Even though *ACS* expression increased at the end of corolla senescence, ethylene production decreased, indicating that ACS activity or stability could be affected by posttranscriptional regulation during corolla senescence.

The early upregulation of PCD-related gene expression supported early PCD in the senescing corollas of *PhATG6*-KO lines. ATG6 is an essential component of the PI3K protein complex in the autophagy pathway (McKnight and Yue, 2013). ATG6 serves as a binding hub for different interacting proteins to regulate the composition, subcellular localization, and/or function of the PI3K complex and consequently affect autophagy activity (He and Levine, 2010; Hill et al., 2019). Therefore, knocking out *ATG6* could result in direct and/or indirect effects on autophagy and other PCD regulatory mechanisms. Our previous study shows that *ATG6* expression is upregulated when *PI3K* is silenced, and *ATG8d* expression is downregulated when *ATG6* is silenced (Lin and Jones, 2021). This study showed that *PhATG6*, *PhPI3K*, and *PhATG8d* expression was upregulated earlier in the *PhATG6*-KO corollas than the controls, correlated with the accelerated senescence (Figures 6A–C). The early induction of these genes was likely a result of the early increase of ethylene production, as ethylene treatment induces autophagy gene expression in petunia and Japanese morning glory (Shibuya et al., 2013; Quijia Pillajo et al., 2018). Apoptosis is another type of PCD modulated by caspases, and the interaction between autophagic PCD and apoptotic PCD is based on caspase-mediated cleavage of ATG6 (Djavaheri-Mergny et al., 2010; Wirawan et al., 2010). In plants, the apoptosis-like PCD is regulated by metacaspases, a group of cysteine proteases similar to mammalian caspases (Minina et al., 2013). RNAi-suppressed *PhMC1* similarly causes accelerated corolla senescence in petunia (Chapin et al., 2017). The expression of *PhMC1* was also upregulated earlier in *PhATG6*-KO lines (Figure 6D), suggesting accelerated PCD in the corollas and indicating a potential interaction between autophagy and metacaspases at the transcriptional level.

Knocking out *PhATG6* in petunia negatively impacted nutrient remobilization during corolla senescence of unpollinated flowers. During leaf senescence, autophagy plays an important role in N remobilization as shown in the atg mutants of Arabidopsis, rice, and maize, where the remobilization efficiency of N is reduced (Guiboileau et al., 2012; Li et al., 2015; Wada et al., 2015; Yu et al., 2019; Fan et al., 2020). Even though the remobilization of N was not affected in the *PhATG6-*KO lines during corolla senescence, P remobilization was decreased in the KO lines (Figures 7A,B). The expression of two petal senescence-specific genes, a cysteine protease *PhCP10* and a phosphate transporter *PhPT1*, was induced earlier in the *PhATG6-*KO lines, and *PhCP10* showed a trend of downregulated expression in the KO plants (Figures 7C,D), indicating that autophagy affects the transcription of genes involved in nutrient degradation and transportation. Cysteine proteases play an important role in protein degradation during petal senescence (Jones et al., 1995; Wagstaff et al., 2002; Arora and Singh, 2004). *PhCP10*, a homolog to Arabidopsis *Senescence-Associated Gene 12* (*SAG12*), is exclusively expressed in senescing petals and its expression is delayed in ethylene-insensitive petunias, corresponding to the delayed corolla senescence (Jones et al., 2005). *PhPT1*, a high-affinity phosphate transporter, belongs to the Phosphate Transporter1 family in petunia (Wegmüller et al., 2008; Chapin and Jones, 2009). Compared to the other petunia high-affinity phosphate transporters, *PhPT1* is highly expressed in senescing petunia corollas (Chapin and Jones, 2009). Ethylene affects the expression of *PhPT1*, as the transcript levels of *PhPT1* decrease in ethylene-insensitive plants during corolla senescence, and ethylene treatment induces *PhPT1* expression in corollas (Chapin and Jones, 2009). The early induction of *PhCP10* and *PhPT1* expression in the *PhATG6*-KO lines was likely the result of the early increased ethylene production. Autophagy may affect nutrient remobilization *via* ethylene-mediated transcriptional regulation of genes involved in nutrient degradation and transportation.

Knocking out a central autophagy gene *PhATG6* in petunia led to accelerated petal senescence, decreased P remobilization, and reduced seed yield, likely due to early expression of senescence-related genes and early ethylene production. Our study contributes to the current understanding of autophagy in senescence and nutrient remobilization and provides information for the improvement of flower longevity in ornamental plants.

## Data Availability Statement

The raw data supporting the conclusions of this article will be made available by the authors, without undue reservation.

## Author Contributions

YL and MLJ conceptualized the project and designed the experiments. YL conducted the experiments and led the writing of the manuscript. MLJ acquired resources and funding for the study, supervised the project, and edited the manuscript. All authors contributed to the manuscript revision and approved the submitted version.

## Funding

The funding for this study was sponsored by the American Floral Endowment, the OSU D.C. Kiplinger Floriculture Endowment, and the OSU Alumni Grants for Graduate Research and Scholarship. Salaries and research support were provided in part by State and Federal funds appropriated to the College of Food, Agricultural and Environmental Sciences, The Ohio State University. Department of Horticulture and Crop Science Manuscript #21-15.

## Conflict of Interest

The authors declare that the research was conducted in the absence of any commercial or financial relationships that could be construed as a potential conflict of interest.

## Publisher’s Note

All claims expressed in this article are solely those of the authors and do not necessarily represent those of their affiliated organizations, or those of the publisher, the editors and the reviewers. Any product that may be evaluated in this article, or claim that may be made by its manufacturer, is not guaranteed or endorsed by the publisher.

## Footnotes

^1^https://solgenomics.net, accessed on 31st March 2021.

^2^https://solgenomics.net, accessed on November 24, 2018.

^3^http://www.rgenome.net/cas-offinder/, accessed on December 17, 2018.

^4^https://web.expasy.org/translate/, accessed on May 6, 2021.
