# Supplementary material for: CRISPR/Cas9-Mediated Editing of Autophagy Gene 6 in Petunia Decreases Flower Longevity, Seed Yield, and Phosphorus Remobilization by Accelerating Ethylene Production and Senescence-Related Gene Expression
Source: Front Plant Sci. 2022 Apr 26;13:840218. doi: 10.3389/fpls.2022.840218 (PMC9088004; doi:10.3389/fpls.2022.840218)
Supplement: Supplementary file 1 [file Data_Sheet_1.pdf]

Supplementary Material

Table 1S. Primers for gene expression analyses

Gene	Forward primer (5' – 3')	Reverse primer (5' – 3')
Actin (*PhActin*) (Chapin and Jones, 2009)	AGCCAACAGAGAGAAGATGACCCA	ACACCATCACCAGAGTCCACCACA
SAND Family Protein (*PhSAND*) (Mallona et al., 2010)	CTTACGACGAGTTCAGATGCC	TAAGTCCTCAACACGCATGC
Autophagy Gene 6 (*PhATG6*) (Lin and Jones, 2021)	CGATGATAAGGGTCGGAGTTTAC	CCGGCGATACAAAGTGGATTA
Phosphoinositide 3-Kinase (*PhPI3K*) (Lin and Jones, 2021)	CTCCCTGTCACTTTCCGTATTG	CATACAGCTCTGGCTTTCTCTC
Autophagy Gene 8d (*PhATG8d*) (Shibuya et al., 2013)	TTGCATGCATCCTCACTCTT	TCAGTTAAAGGGTATAGACAAATCCA
Cysteine Protease 10 (*PhCP10*) (Chapin et al., 2017)	ACTTTGTGGACTTGCAACGGAAGC	CCAAGCCTATCTCAATCCCATACA
Metacaspase 1 (*PhMC1*) (Chapin et al., 2017)	AGGAGCCTCAACTATCCTCGTA	CATGATCAAGGTTCACACAGAA
Phosphate transporter 1 (*PhPT1*) (Chapin and Jones, 2009)	AAGCAATTCCTCCGTCGTCATGGA	TAGCCTGGAACAGTACTGCAGAGA
1-Aminocyclopropane 1-Carboxylate Synthase (*PhACS*) (Lin and Jones, 2021)	GAGAGGAGACTAAGCCAGTAAAG	CAAGTGGTGAGTGAGGTATAGG
1-Aminocyclopropane 1-Carboxylate Oxidase 1 (*PhACO1*) (Lin and Jones, 2021)	AGACGCTGGTGGCATAATCCTTCT	GGTTAACCACAATGGAATGGCGCA

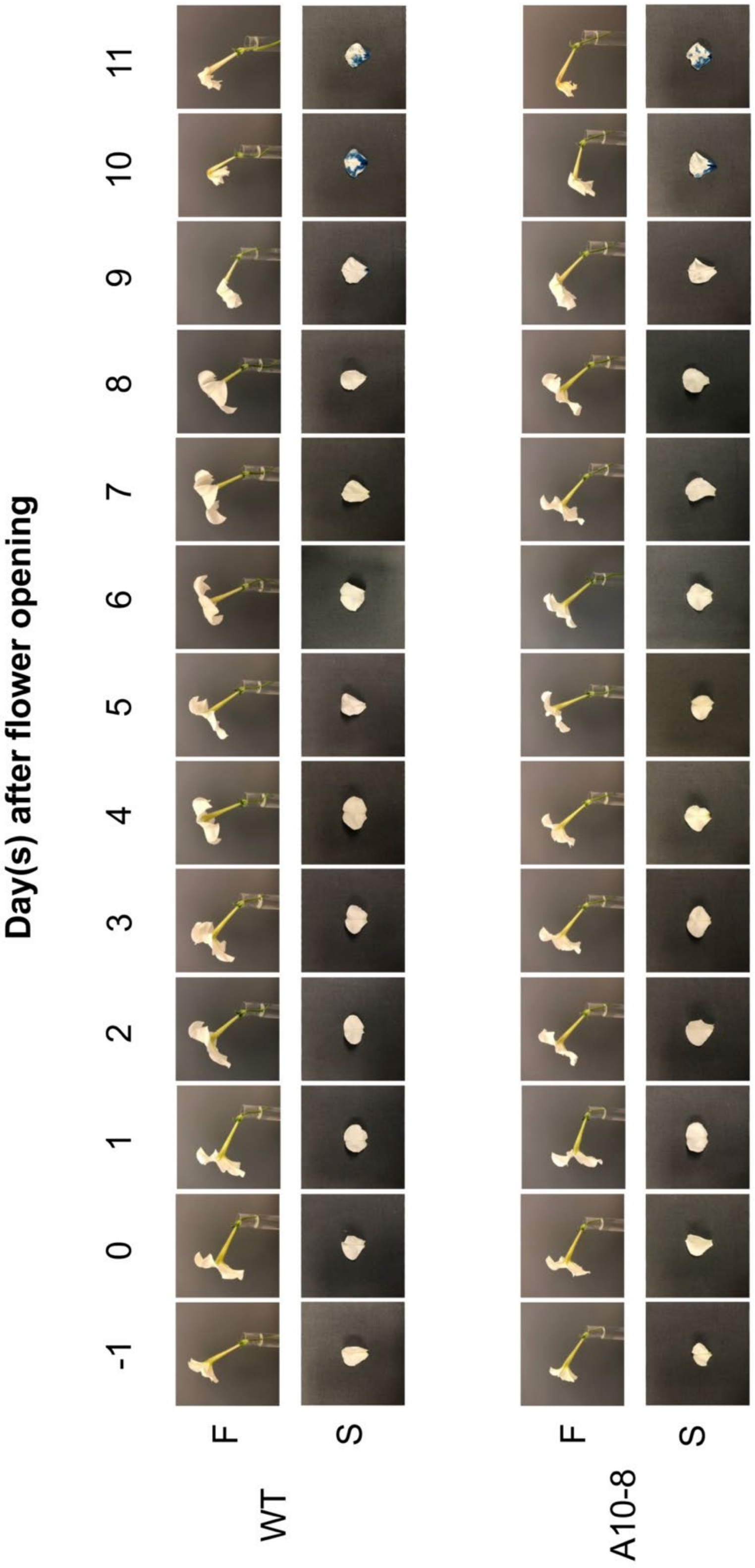

Day(s) after flower opening
-1
0
1
2
3
4
5
6
7
8
9
10
11
WT
F
S
A10-8
F
S

Figure 1S (part 1). Corolla senescence process of Petunia × hybrida ‘Mitchell Diploid’ wild-type plant (WT) and non-mutated regenerated line A10-8. Pictures of representative flowers (F) and Evans Blue-stained petal limbs (S) were taken from the day before flower opening (day -1) to the day after flower senescence.

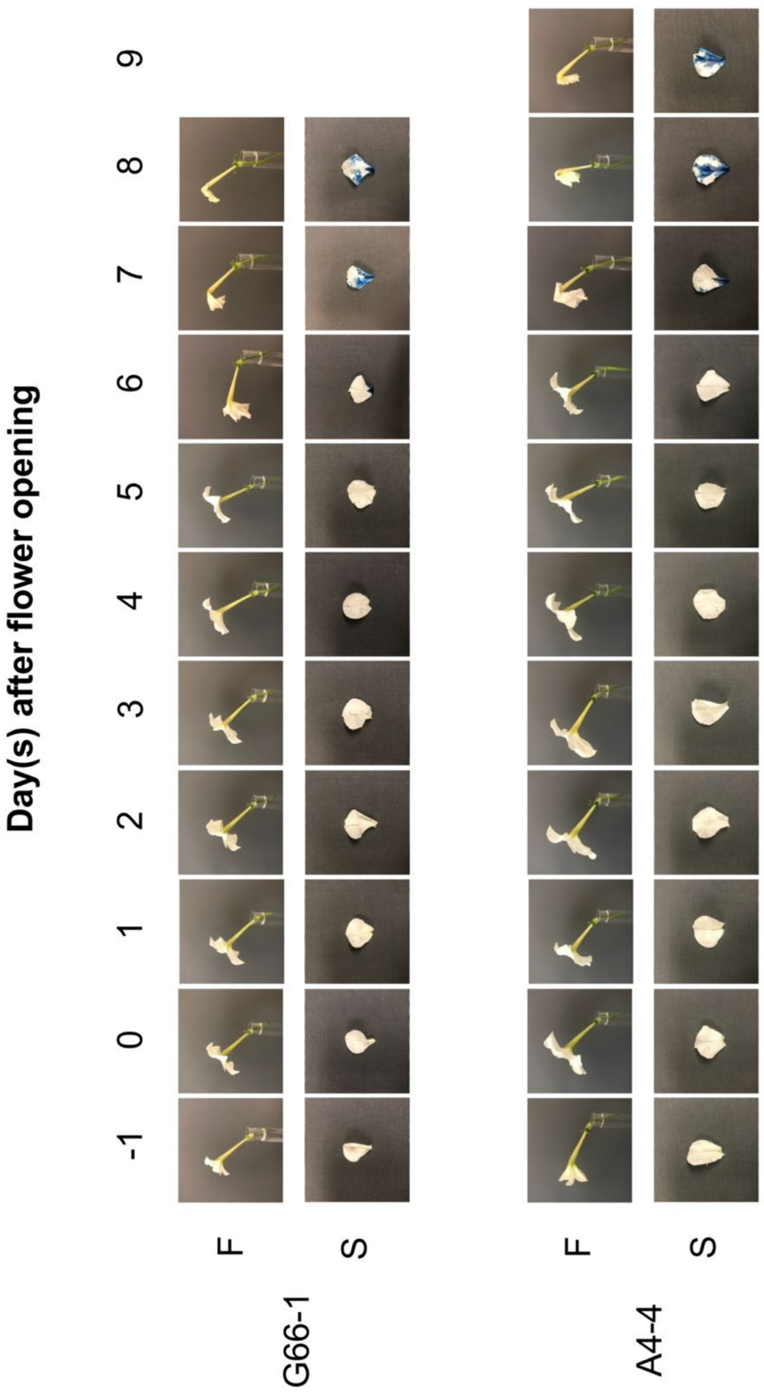

Day(s) after flower opening
-1
0
1
2
3
4
5
6
7
8
9
G66-1
F
S
A4-4
F
S

Figure 1S (part 2). Corolla senescence process of Petunia × hybrida 'Mitchell Diploid' *PhATG6*-KO lines (G66-1 and A4-4). Pictures of representative flowers (F) and Evans Blue-stained petal limbs (S) were taken from the day before flower opening (day -1) to the day after flower senescence.

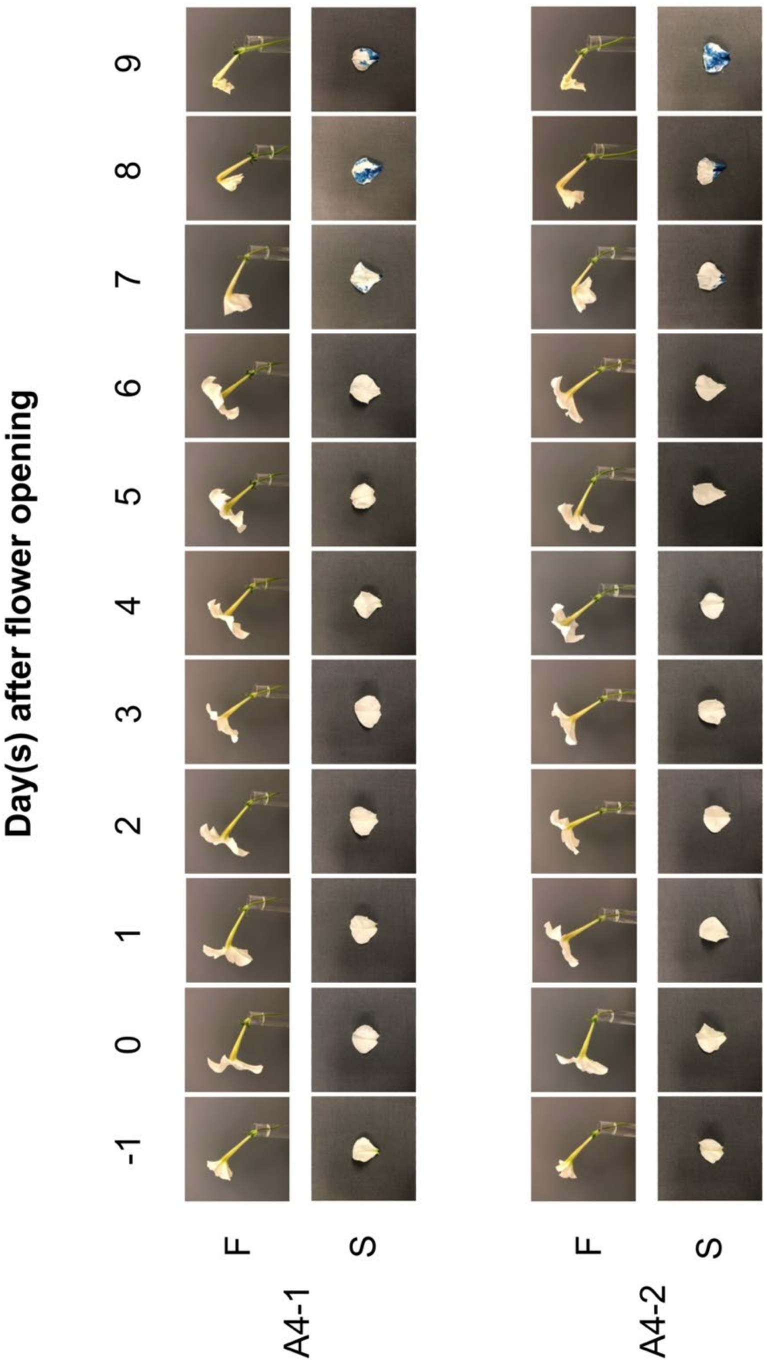
Day(s) after flower opening
-1
0
1
2
3
4
5
6
7
8
9
A4-1
F
S
A4-2
F
S

Figure 1S (part 3). Corolla senescence process of Petunia × hybrida 'Mitchell Diploid' *PhATG6*-KO lines (A4-1 and A4-2). Pictures of representative flowers (F) and Evans Blue-stained petal limbs (S) were taken from the day before flower opening (day -1) to the day after flower senescence.

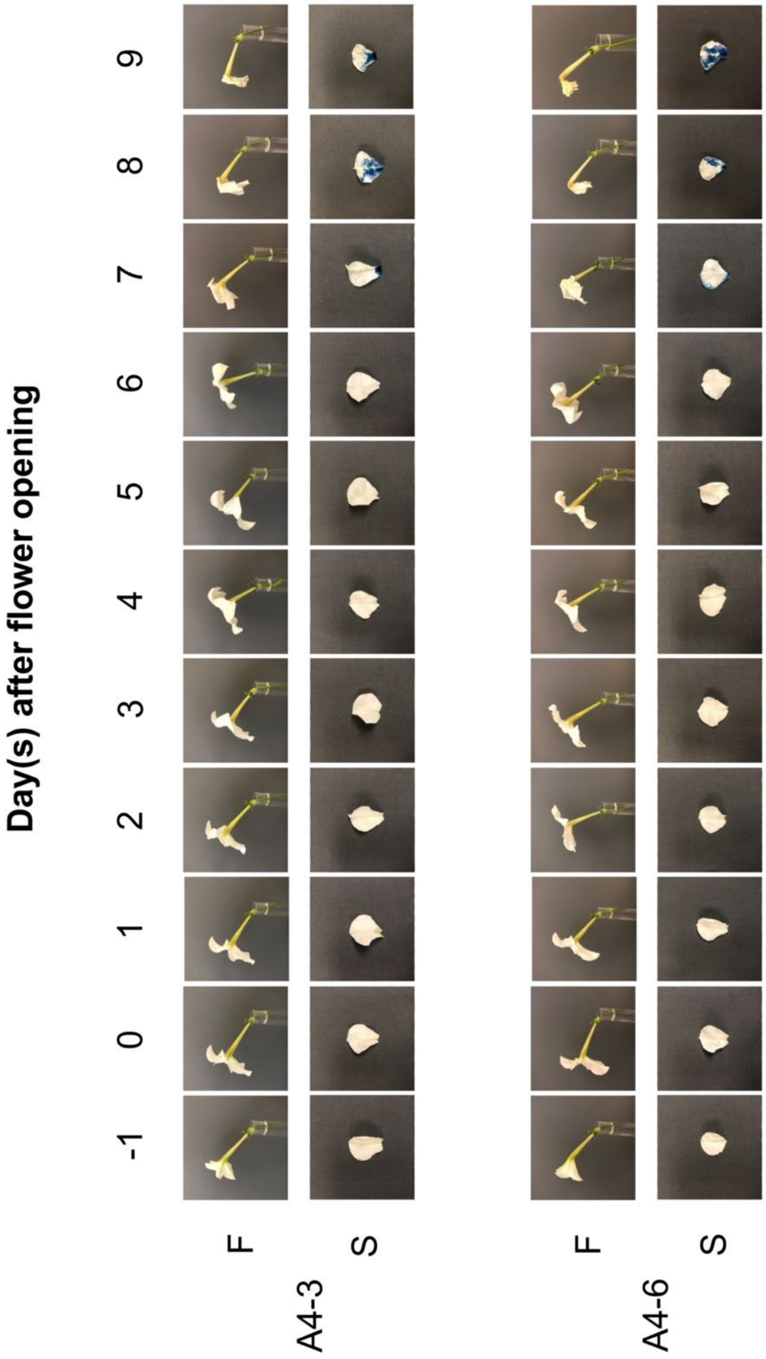

Day(s) after flower opening
-1
0
1
2
3
4
5
6
7
8
9
A4-3
F
S
A4-6
F
S

Figure 1S (part 4). Corolla senescence process of Petunia × hybrida ‘Mitchell Diploid’ *PhATG6*-KO lines (A4-3 and A4-6). Pictures of representative flowers (F) and Evans Blue-stained petal limbs (S) were taken from the day before flower opening (day -1) to the day after flower senescence.
